# Using the Natural Language Processing System Medical Named Entity Recognition-Japanese to Analyze Pharmaceutical Care Records: Natural Language Processing Analysis

**DOI:** 10.2196/55798

**Published:** 2024-06-04

**Authors:** Yukiko Ohno, Riri Kato, Haruki Ishikawa, Tomohiro Nishiyama, Minae Isawa, Mayumi Mochizuki, Eiji Aramaki, Tohru Aomori

**Affiliations:** 1 Faculty of Pharmacy Keio University Tokyo Japan; 2 Nara Institute of Science and Technology Nara Japan; 3 Faculty of Pharmacy Takasaki University of Health and Welfare Gunma Japan

**Keywords:** natural language processing, NLP, named entity recognition, pharmaceutical care records, machine learning, cefazolin sodium, electronic medical record, EMR, extraction, Japanese

## Abstract

**Background:**

Large language models have propelled recent advances in artificial intelligence technology, facilitating the extraction of medical information from unstructured data such as medical records. Although named entity recognition (NER) is used to extract data from physicians’ records, it has yet to be widely applied to pharmaceutical care records.

**Objective:**

In this study, we aimed to investigate the feasibility of automatic extraction of the information regarding patients’ diseases and symptoms from pharmaceutical care records. The verification was performed using Medical Named Entity Recognition-Japanese (MedNER-J), a Japanese disease-extraction system designed for physicians’ records.

**Methods:**

MedNER-J was applied to subjective, objective, assessment, and plan data from the care records of 49 patients who received cefazolin sodium injection at Keio University Hospital between April 2018 and March 2019. The performance of MedNER-J was evaluated in terms of precision, recall, and *F*_1_-score.

**Results:**

The *F*_1_-scores of NER for subjective, objective, assessment, and plan data were 0.46, 0.70, 0.76, and 0.35, respectively. In NER and positive-negative classification, the *F*_1_-scores were 0.28, 0.39, 0.64, and 0.077, respectively. The *F*_1_-scores of NER for objective (0.70) and assessment data (0.76) were higher than those for subjective and plan data, which supported the superiority of NER performance for objective and assessment data. This might be because objective and assessment data contained many technical terms, similar to the training data for MedNER-J. Meanwhile, the *F*_1_-score of NER and positive-negative classification was high for assessment data alone (*F*_1_-score=0.64), which was attributed to the similarity of its description format and contents to those of the training data.

**Conclusions:**

MedNER-J successfully read pharmaceutical care records and showed the best performance for assessment data. However, challenges remain in analyzing records other than assessment data. Therefore, it will be necessary to reinforce the training data for subjective data in order to apply the system to pharmaceutical care records.

## Introduction

### Background

Natural language processing (NLP) is a computer technology to process the language people read and write in their daily lives. Machine translation and search engines are examples of NLP technologies.

In recent years, with advancements in artificial intelligence technology, it has become possible to extract information related to patients’ diseases and symptoms from unstructured data such as medical records [1,2].

NLP technology that is used to extract information such as that related to diseases and symptoms, the names of people and organizations, time expressions, and numerical expressions from text is generally referred to as named entity recognition (NER). Some NER systems also have a positive-negative (P-N) classification function that can be used to determine the onset of extracted findings.

To date, most research on NLP technology has focused on English-language texts. NLP technology that is focused on Japanese texts has lagged due to certain aspects of the Japanese language, including that words are not separated by spaces and subjects are often omitted [3].

### Related Studies

Among Japanese NLP studies that focused on medical issues, the study by Imai et al [4] developed a system that performs extraction and P-N classification of malignant findings from radiological reports such as computed tomography reports and magnetic resonance imaging reports; Ma et al [5] built a system that performs extraction and P-N classification of abnormal findings from discharge summaries, progress notes, and nursery notes; and Aramaki et al [6] developed a system that performs extraction and P-N classification of disease names and symptoms from case history summaries. In addition, Mashima et al [7] extracted adverse events from progress notes of patients who received intravenous injections of cytotoxic anticancer drugs, and Usui et al [8] extracted symptomatic states from data stored in the electronic medical records of a community pharmacy and standardized them according to the codes of the *ICD-10* (*International Classification of Diseases, Tenth Revision*) in order to create a data set of patients’ complaints [9,10]. The National Institute of Informatics Test beds and Community for Information Access Research Project’s *Medical Natural Language Processing for Web Document* task aimed to classify pseudotweets according to whether they contained information about patients’ symptoms [9], and several teams collaborated to build a system to accomplish this task. Nishioka et al [10] established a system to identify from blog posts whether a patient is positive or negative for hand-foot syndrome on a per-patient and per-sentence basis. Although various approaches have been taken to analyze unstructured medical data as described above in this section, most have targeted physicians’ records, including case history summaries, discharge summaries, and radiological reports, and NER has not been widely applied to pharmaceutical care records.

Pharmaceutical care records are documents about patients and are written by pharmacists, who collect information from a pharmacological perspective. Because pharmaceutical care records contain an entry for the change in patients’ physical condition while taking medication, including symptoms of suspected adverse drug effects [11], many such symptoms are documented in pharmaceutical care records. Thus, developing an NER system that can extract and analyze information from pharmaceutical care records would facilitate investigations of adverse drug effects.

The study by Usui et al [8], mentioned in the beginning of this section, targeted data similar to this study. Because their system was a rule-based model, it had difficulty handling symptoms and contexts that were not set in the rules. Although rules can be added, it is difficult to manage them with consistency. Therefore, we aimed to overcome this problem by using machine learning.

### Study Aim

In this study, we applied Medical Named Entity Recognition-Japanese (MedNER-J), a Japanese-language system designed to extract disease information from physicians’ records [6], to pharmaceutical care records in order to verify the feasibility of NER and P-N classification for this task. Target data were pharmaceutical care records of patients who received cefazolin sodium (CEZ) injection. CEZ is a cephem antibiotic that is often used to prevent secondary infection from operative wounds. The system was applied only to the records of patients who received CEZ injection, with the expectation of mainly collecting target drug information due to fewer concomitant drugs.

This is the first study to apply the existing system to pharmaceutical care records. This study provides the baseline performance for analyzing Japanese pharmaceutical care records using the machine learning method.

## Methods

### Materials

**Figure 1:**
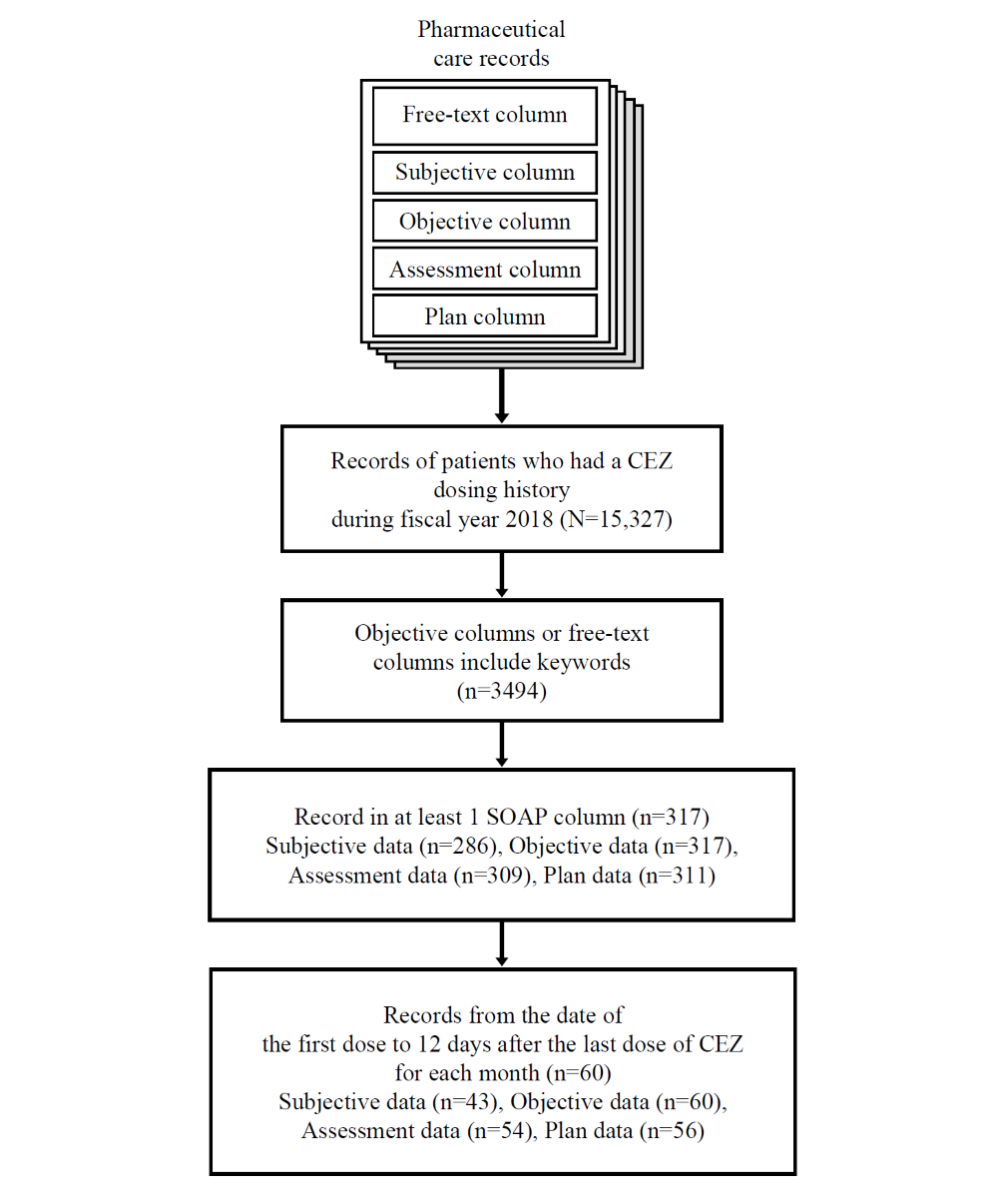
Data set preparation. Among the records from April 2018 to March 2019, those from the date of first cefazolin sodium (CEZ) administration to 12 days after the end of administration that also contained the keywords in the objective column or the free-text column and a record in one of the subjective, objective, assessment, and plan (SOAP) columns were included in the analysis.

Pharmaceutical care records of patients who received CEZ injection between April 2018 and March 2019 at Keio University Hospital were used as test data (Figure 1). Researchers accessed and obtained those data on November 19, 2021.

Pharmaceutical care records were written by pharmacists, and the format consisted of (1) free-text columns and (2) subjective, objective, assessment, and plan (SOAP) columns: subjective information such as patients’ complaints were included in the subjective data; objective information such as clinical history, clinical findings, and laboratory data were included in the objective data; assessments by pharmacists were included in the assessment data; and future plans were included in the plan data.

Data that satisfied the following criteria were used in this research: (1) records with a description in at least 1 SOAP column and (2) records including any of the following keywords in the free-text column or objective column: *cefazolin* (written in full-width or half-width katakana characters), *cefamezin* (written in full-width or half-width katakana characters), *CEZ*, and *cez*.

MedNER-J was applied to the records that satisfied the above criteria and that corresponded to the period from the first CEZ dosing day to 12 days after the last dosing for each patient for each month.

### NER and P-N Classification

**Figure 2:**
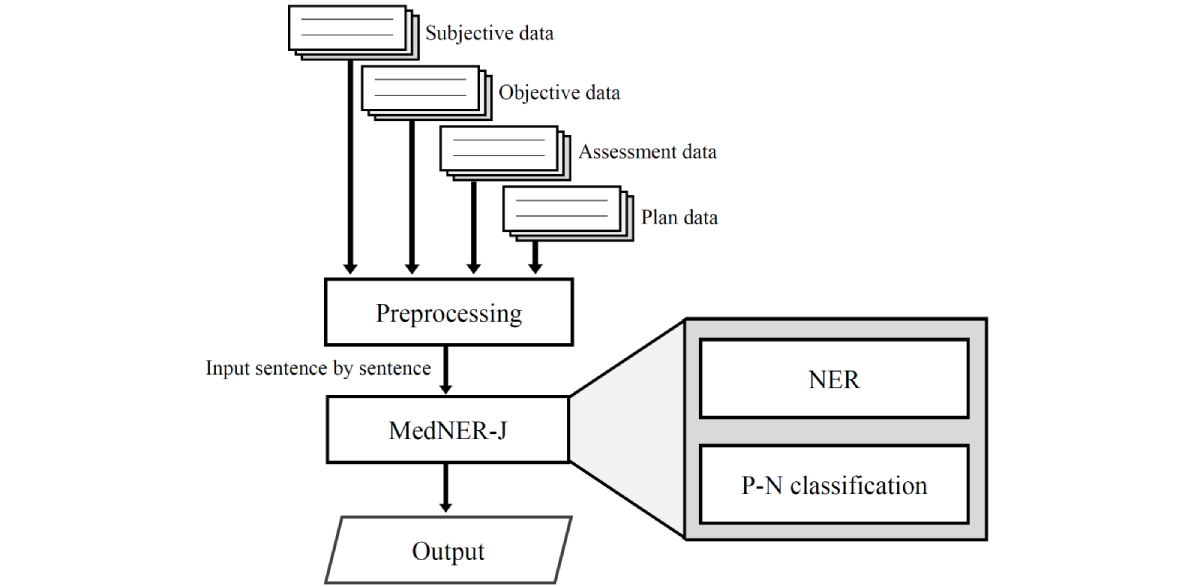
Processing of pharmaceutical care records. Each subjective, objective, assessment, and plan (SOAP) column underwent preprocessing as well as named entity recognition (NER) and positive-negative (P-N) classification by Medical Named Entity Recognition-Japanese (MedNER-J) to obtain the final results.

We used MedNER-J [12] for NER and P-N classification (Figure 2). MedNER-J is an NLP system to extract information related to diseases and symptoms from physicians’ records. It is based on the bidirectional encoder representations from transformers (BERT) [13]–conditional random fields (CRF) model [14], which fine-tuned case history summaries to pretrained Japanese BERT model established by Tohoku NLP group [15]. CRF was used in a previous NER task [6]; however, BERT-CRF performed better than CRF [16]. In addition, MedNER-J can extract data related to a wide range of diseases and symptoms although many systems in previous studies targeted specific diseases and symptoms. Therefore, we used MedNER-J for this study. The system can perform P-N classification in order to determine the onset or absence of presumed findings from the context.

At the preprocessing stage, all characters in the records were converted to full-width characters, and exclamation marks were converted to periods.

Preprocessed records were input to MedNER-J on a sentence-by-sentence basis to perform NER and P-N classification. A sentence break was defined as a line break or a period.

### Performance Evaluation

**Figure 3:**
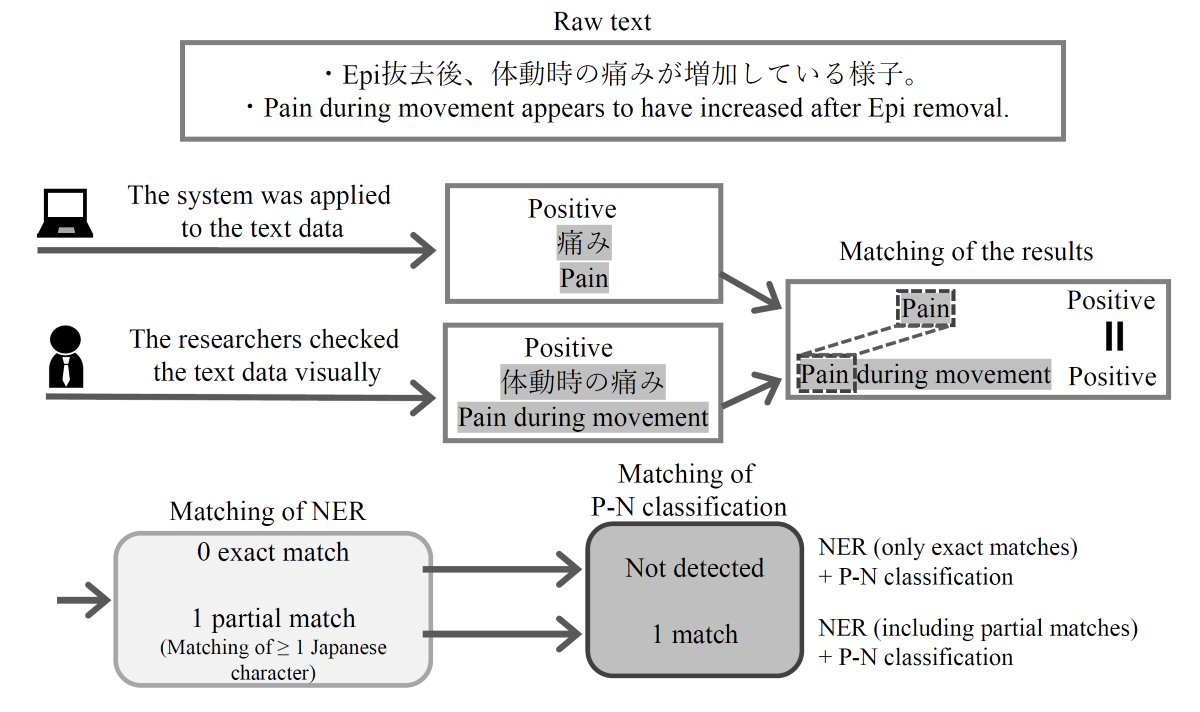
Flow of result matching. The system’s results were matched with the researchers’ results, and performance evaluation indexes were calculated based on the number of named entity recognition (NER) matches alone and the number of NER and positive-negative (P-N) classification matches. Both exact matches as well as partial matches were obtained for NER. Epi: epidural anesthesia.

Figure 3 shows the performance evaluation flow. A total of 2 researchers independently extracted named entities from the same records, performed P-N classification by visual confirmation, and created the correct answer data following the original criteria. Exact and partial matches of extracted terms between MedNER-J and the 2 researchers were examined, and P-N classification matches were also investigated. The criteria the researchers followed to create the correct answer data will be explained in the *Judging Criteria for Researchers* section.

In cases where 1 sentence contained the same named entities multiple times, researchers also checked whether the positional relationships in the sentence were matched for the same extracted named entities. If the extracted terms matched exactly, they were judged as exact matches. In cases where they did not match exactly but overlapped by ≥1 Japanese characters, they were judged as partial matches. Both exact match extractions and partial match extractions were checked in terms of P-N classification.

Precision, recall, and *F*_1_-score were calculated and evaluated for the following: matches of NER (including partial matches), and matches of NER in addition to P-N classification results (including partial matches).

Precision = number of true positive/number of true positive and false positive **(1)**

Recall = number of true positive/number of true positive and false negative **(2)**

*F*_1_-score = (2 × precision × recall)/(precision + recall) **(3)**

When counting the results, including partial matches, the number of matched terms varied depending on whether they were counted in units of the researchers’ extracted terms or in units of the system’s extracted terms. In such cases, counts were made according to the units that reduced the total number of matched terms.

The validity of researchers’ evaluations was examined using κ coefficients [17]. Mismatched results between 2 researchers were discussed, and judgment results between the researchers were adjusted to be reasonable. The κ coefficient of the 2 researchers was 0.87, indicating a high degree of concordance; this showed that researchers’ evaluations were appropriate.

The mismatched results between MedNER-J and the researchers were categorized as follows: (1) system extraction failure, (2) incorrect extraction by the system, (3) difference in P-N classification, and (4) difference in the length of the extracted terms. The number of mismatched terms also varied depending on whether they were counted in units of terms extracted by the system or terms extracted by the researchers. In such cases, counts were made according to units that increased the number of mismatched terms.

After categorization, the features of mismatched terms in each category were explored, with the aim of understanding what the system was then incapable of doing and discussing how those features had affected the analyses performed by the system.

### Judging Criteria for Researchers

This section outlines the criteria that the researchers used to create the correct answer data. Not only nouns such as *pain* but also verbs such as *hurt*, adjectives such as *sore*, and adverbs such as *painfully* were considered targets for extraction. Symptom modifiers such as site, timing, and severity of symptom onset were also considered together with the symptoms to be extracted. With regard to terms meaning patients’ conditions such as *sleep*, *appetite*, *state of bowel movements*, *renal function*, *hepatic function*, and *blood electrolyte levels*, if only a statement of normality such as “appetite is fine” was given, it was also considered to be a target for extraction. For example, pharmacists often ask patients whether they have experienced a loss of appetite, and patients’ responses such as “appetite is fine” are recorded frequently. Such normal states were difficult to consider as diseases or symptoms. Though targets of extraction for records analysis were diseases and symptoms, they are also considered to be important information about patients. Therefore, the terms indicating these six conditions were considered for extraction by the researchers. English abbreviations other than laboratory values were consistently excluded from extraction by the researchers. This is because some of them have different meanings among different medical departments, and it was difficult to use the extracted terms by themselves. Laboratory values and vital signs were considered for extraction only if words or symbols clearly stated the numerical change or how it was abnormal, with the exceptions of *renal function*, *hepatic function*, and *blood electrolyte concentration*. If only numerical information on laboratory values and vital signs were provided, the information was excluded from extraction because this information is obtainable from the structured data of the medical records, and thus there is no need to extract it from the text data. When symptoms were described consecutively, each symptom was considered as an individual symptom. For *allergy*, any modifiers that indicate the types of allergies listed in the medical dictionary for regulatory activities (MedDRA) were also considered for extraction. For example, if there was a description of “allergy caused by a drug,” this could be classified as “drug hypersensitivity” in MedDRA. Therefore, the modifier “caused by a drug” was included in the extracted data. In some cases, specific drug names were mentioned, for example, the description “allergy caused by cefazolin.” However, the drug name “cefazolin” does not appear in MedDRA. If a drug name that does not appear in MedDRA was included in description, only *allergy* was considered as an extraction target, and any modifiers were excluded. Although the description “medication for diseases (eg, diabetes)” was also included, it was not possible to determine whether the medication was used for the patients themselves. Therefore, “diseases (diabetes)” in “medication for diseases (diabetes)” was excluded from extraction. “Symptom (eg, pain)” in “symptom (pain) monitoring” was excluded from extraction because that symptom could not be detected in terms of onset or absence.

In the P-N classification process, the researchers considered symptoms that were currently present in the patients themselves as positive symptoms in principle. The onset and absence of symptoms were determined by referring only to the context within a given sentence. Use of medication to be taken as needed, such as “times of pain,” was regarded as a negative symptom because onset had not yet occurred. Adverse drug effects mentioned in the explanation of the drug used were considered to be negative symptoms because they did not actually occur. Past symptoms that were not stated to have resolved, such as “I couldn’t sleep last night,” were considered to be positive symptoms. If there was even a slight improvement in symptoms, they were considered to be negative symptoms. Other cases in which the onset of symptoms could not be determined were considered to be positive symptoms.

### Ethical Considerations

This study was approved by the Keio University School of Medicine ethics committee (approval number 2020067). The researchers used only record data that have been previously deidentified by removing patient names and replacing real patient IDs with dummy IDs. Only the personal information manager, who was not included in the authors, had access to the correspondence table between the real patient ID and the dummy ID. A written opt-out form was implemented instead of informed consent. The opt-out document is available from the website of Keio University Hospital.

## Results

### Target Records Features

Of the 15,327 records of patients who received CEZ injection during the 2018 fiscal year, 317 (2.07%) pharmaceutical care records satisfied both the inclusion criteria (Figure 1). The number of records (n=60) obtained within the period following CEZ injection were 43 (72%) for subjective data (38 patients), 60 (100%) for objective data (49 patients), 54 (90%) for assessment data (45 patients), and 56 (93%) for plan data (46 patients). The total number of sentences contained in the records obtained decreased in the following order: objective data>assessment data>subjective data>plan data (Table 1). The median value of characters per sentence decreased in the following order: objective data>subjective data>assessment data>plan data (Table 1).

**Table 1:** Features of target records (number of sentences and characters).

			Subjective data		Objective data		Assessment data		Plan data
**Sentences (n=3090)**									
	Total, n (%)	211 (6.83)		2431 (78.7)		338 (10.9)		110 (3.56)	
	Median (per record)	4		37		4.5		2	
	Maximum value (per record)	17		120		21		5	
	Minimum value (per record)	1		5		2		1	
**Characters (n=85,824)**									
	Total, n (%)	4378 (5.10)		72,991 (85.0)		7125 (8.30)		1330 (1.55)	
	Median (per sentence)	18		29		15		10	
	Maximum value (per sentence)	61		113		97		44	
	Minimum value (per sentence)	1		1		3		1	

### NER and P-N Classification

The number of the records analyzed and the extraction results are shown in Table 2.

Table 3 shows the results of the performance evaluation. The recall of subjective and assessment data was lower than precision for both NER alone and for NER and P-N classification. Precision was higher than recall for plan data. Recall was similar to precision for objective data.

A trade-off relationship exists between precision and recall, meaning that when one increases, the other decreases. Therefore, the *F*_1_-score, which is the harmonic mean of precision and recall, is used as an evaluation index for overall performance. The results of *F*_1_-score in Table 3 show that MedNER-J was able to conduct NER and P-N classification with high performance in the following order: assessment data>objective data>subjective data>plan data.

**Table 2:** Number of the records analyzed and extraction results by the MedNER-J^a^ system and researchers.

		Subjective data, n (%)	Objective data, n (%)	Assessment data, n (%)	Plan data, n (%)
Records analyzed (n=60)		43 (71.7)	60 (100)	54 (90)	56 (93.3)
**Extracted terms**					
	System (n=633)	50 (7.9)	411 (64.9)	135 (21.3)	37 (5.8)
	Researchers (n=805)	130 (16.1)	444 (55.2)	216 (26.8)	15 (1.9)
**Matches**					
	NER^b,c^ (n=483)	41 (8.5)	300 (62.1)	133 (27.5)	9 (1.9)
	NER^c^ +P-N^d^ classification (n=305)	25 (8.2)	165 (54.1)	113 (37)	2 (0.7)

^a^MedNER-J: Medical Named Entity Recognition-Japanese.

^b^NER: named entity recognition.

^c^Including partial matches.

^d^P-N: positive-negative.

**Table 3:** Performance evaluation of named entity recognition (NER) and positive-negative (P-N) classification.

		Precision	Recall	*F*_1_-score
**NER (including partial matches)**				
	All data	0.76	0.60	0.67
	Subjective data	0.82	0.32	0.46
	Objective data	0.73	0.68	0.70
	Assessment data	0.99	0.62	0.76
	Plan data	0.24	0.60	0.35
**NER (including partial matches)+P-N classification**				
	All data	0.48	0.38	0.42
	Subjective data	0.50	0.19	0.28
	Objective data	0.40	0.37	0.39
	Assessment data	0.84	0.52	0.64
	Plan data	0.054	0.13	0.077

### Error Analysis

Table 4 shows the categories of causes of mismatches between the system and the researchers.

As each type of SOAP data contained differing amounts of information about diseases and symptoms, a comparison of mismatch causes between these data should be based on the percentage of mismatched terms out of the total extracted terms (sum of the number of extracted terms by the system and the researchers−number of matched terms), not the number of mismatched terms. In the calculation of this percentage, partial matches were considered matches in system extraction failure (cause category 1), incorrect extraction by the system (cause category 2), and difference in P-N classification (cause category 3), while partial matches were considered mismatches in cause category 4. Therefore, the percentage of system extraction failure (cause category 1), incorrect extraction by the system (cause category 2), difference in P-N classification (cause category 3), and difference in the length of the extracted terms (cause category 4) does not add up to 100%. Comparing the percentages, the largest percentage of mismatches was subjective data (89/139, 64%) in cause category 1, plan data (28/43, 65%) in cause category 2, objective data (138/555, 24.9%) in cause category 3, and objective data (81/555, 14.6%) in cause category 4.

The researchers classified terms in the 4 cause categories shown in Table 4 into subcategories according to the features of the mismatched term itself and the context around the mismatched term. If a mismatched term had multiple features, it was counted in >1 subcategory.

The subjective and assessment data were expected to contain a large amount of adverse drug effect information due to the characteristics of the SOAP format. The researchers focused on subjective and assessment data because they expected that the analysis of pharmaceutical care records would facilitate the collection and analysis of information on adverse drug effects. Given that the performance for subjective data was low, in Table 5, we listed the top 5 subcategories that had the highest number of eligible cases in cause category 1, with the highest percentage of mismatches in the subjective data.

The common mismatches in cause category 1 “system extraction failure” were “verbs, adjectives, and adverbs”; “expressions that are difficult to grasp as diseases or symptoms”; and “lists of dosages (medication to be taken as needed; eg, at times of the symptoms).” The most common mismatches in the subjective data were “verbs, adjectives, and adverbs.”

In mismatches of “verbs, adjectives, and adverbs,” many expressions were general terms or colloquialisms that could be included in the patients’ speech, such as “sore” and “I couldn’t sleep.” The mismatches of “expressions that are difficult to grasp as diseases or symptoms” corresponded to expressions such as “bowel movements are fine.” Although they characterized a normal status, they were important for understanding the patient’s health status. “Lists of dosages (medication to be taken as needed) (eg, times of the symptoms)” was a description of the dosage of the medication to be taken as needed.

**Table 4:** Percentage of mismatched terms out of the total number of extracted terms^a^ and the number of mismatched terms in each cause category.

Cause category	Subjective data (n=139)^b^, n (%)	Objective data (n=555)^b^, n (%)	Assessment data (n=218)^b^, n (%)	Plan data (n=43)^b^, n (%)
(1) System extraction failure	89 (64)	139 (25)	83 (38.1)	5 (11.6)
(2) Incorrect extraction by the system	9 (6.5)	111 (20)	2 (0.9)	28 (65.1)
(3) Difference in P-N^c^ classification	16 (11.5)	138 (24.9)	20 (9.2)	8 (18.6)
(4) Difference in the length of the extracted terms	13 (9.4)	81 (14.6)	30 (13.8)	2 (4.7)

^a^Total number of extracted terms = (number of extracted terms by the system + number of extracted terms by researchers) – number of matched terms between the system and researchers (exact matches and partial matches).

^b^Total number of terms extracted from each subjective, objective, assessment, and plan data.

^c^P-N: positive-negative.

**Table 5:** Example breakdown of cause category 1 “system extraction failure.”

Subcategory and example given in Japanese (English)			Scope of the researchers’ extraction, Japanese (English)		Subjective data, n (%)		Objective data, n (%)		Assessment data, n (%)		Plan data, n (%)
**Verbs, adjectives, and adverbs (n=86)**					50 (58.1)		23 (26.7)		13 (15.1)		0 (0)
	まだ痛いですね。 (still hurts)	痛い (hurts)		1 (1.2)		0 (0)		0 (0)		0 (0)	
	昨晩はベルソムラを服用しませんでしたが、眠れなかったそうです。(although he didn’t take Belsomra last night, he couldn’t sleep)	眠れなかった (couldn’t sleep)		0 (0)		1 (1.2)		0 (0)		0 (0)	
	また、腎機能も悪く薬剤の投与量に関しては注意必要 (also, kidney function is poor and drug dosage needs to be carefully monitored)	腎機能も悪く(kidney function is poor)		0 (0)		0 (0)		1 (1.2)		0 (0)	
**Expressions that are difficult to grasp as diseases or symptoms (n=32)**					8 (25)		4 (12.5)		20 (62.5)		0 (0)
	腎機能、肝機能問題なし (kidney function and liver function are fine)	腎機能、肝機能 (kidney function and liver function)		0 (0)		0 (0)		4 (12.5)		0 (0)	
	電解質問題なし (electrolytes are fine)	電解質 (electrolytes)		0 (0)		0 (0)		5 (15.6)		0 (0)	
	今は睡眠について困ってないです。 (I have no trouble sleeping now)	睡眠 (sleeping)		1 (3.1)		0 (0)		0 (0)		0 (0)	
	お通じは1日1回は出ているので問題ないと思います。 (I have a bowel movement once a day, so I think I’m doing OK)	お通じ (bowel movement)		1 (3.1)		0 (0)		0 (0)		0 (0)	
**Lists of dosages (medication to be taken as needed; eg, at times of symptoms; n=20)**					0 (0)		20 (100)		0 (0)		0 (0)
	ロゼレム(8 mg) 1 T 不眠時 1日1回まで (Rozerem, 8 mg, 1 T time of insomnia up to once a day)	不眠 (insomnia)		0 (0)		2 (10)		0 (0)		0 (0)	
	用法 指示簿参照(疼痛時) (use: refer to the instruction manual [time of pain])	疼痛 (pain)		0 (0)		2 (10)		0 (0)		0 (0)	
**Linguistic representation of laboratory values (n=26)**					0 (0)		9 (34.6)		17 (65.4)		0 (0)
	INR^a^短い (INR is short)	INR短い (INR is short)		0 (0)		0 (0)		1 (3.85)		0 (0)	
	WBC^b^とCRP^c^減少傾向を確認した。 (decreasing trends in WBC and CRP were observed)	WBCとCRP減少傾向 (decreasing trends in WBC and CRP)		0 (0)		0 (0)		1 (3.85)		0 (0)	
	L/D^d^より入院時高値だったKは正常値まで低下した。(from L/D, the potassium level, which was high on admission, decreased to normal)	高値だったK (potassium level, which was high)		0 (0)		0 (0)		1 (3.85)		0 (0)	
**Item names (n=13)**					0 (0)		13 (100)		0 (0)		0 (0)
	＜副作用＞ (＜adverse drug effects＞)	副作用 (adverse drug effects)		0 (0)		2 (15.4)		0 (0)		0 (0)	
	副作用： アレルギー、肝機能障害、冷汗など (adverse drug effects: allergy, impaired liver function, cold sweat)	副作用 (adverse drug effects)		0 (0)		1 (7.7)		0 (0)		0 (0)	
	副作用）アナフィラキシーショック,皮膚症状,消化器症状等の副作用の可能性。(adverse drug effects: possibility of adverse drug effects such as anaphylactic shock, skin symptoms, and digestive symptoms)	副作用 (adverse drug effects)		0 (0)		3 (23.1)		0 (0)		0 (0)	

^a^INR: international normalized ratio.

^b^WBC: white blood cell.

^c^CRP: C-reactive protein.

^d^L/D: laboratory data.

## Discussion

### Principal Findings

Our results showed that when MedNER-J was applied to pharmaceutical care records, NER and P-N classification could successfully be performed. However, the performance of the system differed for each type of SOAP data, and some issues remain for practical use. Furthermore, cases in which the system performed inadequately were identified by the analysis of mismatch cause categories.

### Application to Pharmaceutical Care Records

The number of extracted terms by both the system and the researchers were greater in the following order: objective>assessment>subjective> plan data.

The pharmaceutical care records that were targeted in this study included an average of 13.4 (SD 9.3) diseases or symptoms per record. From these records, MedNER-J correctly extracted an average of 8.1 (SD 6.0) terms and correctly extracted and performed P-N classification on an average of 5.1 (SD 4.2) terms. Therefore, MedNER-J was able to extract 60% (8.1/13.4) of findings from the pharmaceutical care records and correctly classify 63% (5.1/8.1) of those findings as positive or negative.

### Performance Evaluation

In this study, we focused on results that included not only exact matches but also partial matches between MedNER-J and the researchers. Word segments in Japanese are unclear, and the necessary extraction range of words varies depending on the situation and the reader. As an example of variations, for the term *itakute* (“in pain”), it is sufficient to extract *itaku* or it may be necessary to extract *itakute*, including the conjunctive particle *te*. In addition, we considered whether expressions related to severity should also be extracted. We speculated that enough information would be extracted from partial matches to ascertain diseases and symptoms. Therefore, we decided to include partial matches and then analyze the results.

Although the *F*_1_-scores for all data were 0.67 for NER alone and 0.42 for NER and P-N classification, values varied among the SOAP data. This variation indicates that the applicability of the system differs for each data set. The *F*_1_-scores of NER for the objective (0.70) and assessment data (0.76) was high, while those of NER for the subjective and plan data were only 0.46 and 0.35, respectively. This indicates that the NER performance for the objective and assessment data was superior to that for the subjective and plan data. At the same time, the *F*_1_-score of NER and P-N classification was high only for assessment data (*F*_1_-score=0.64).

The training data for MedNER-J consisted of case history summaries. Because machine learning systems are generally optimized for the analysis of the training data, the system was optimized for the analysis of case history summaries. Case history summaries include chief complaints, medical history, laboratory findings, and discussions of each case, as summarized by physicians. Thus, in case history summaries, unlike the pharmaceutical care records written in the SOAP format, the patients’ raw statements in the subjective data could have been replaced by the physicians’ expressions. In addition, the plan data used in this study contained only 15 terms of symptoms, and many records ended with brief descriptions such as “observe the progress.” These points are considered to differ from case history summaries, which describe follow-up plan along with the discussion. This might have resulted in lower performance for the subjective and plan data. In contrast, the objective and assessment data were written in the pharmacists’ expressions and described diseases and symptoms in technical terminology, which likely contributed to the high NER performance. Moreover, “progress and discussion of the disease” are a requisite part of case history summaries [18], and this point was similar to the description of the assessment data. This is probably why the *F*_1_-score including P-N classification for the assessment data was high. A decrease in recall implies an increase in false negatives, while a decrease in precision implies an increase in false positives. Therefore, the lower recall compared to precision for the subjective and assessment data indicate that many mismatches were due to cause category 1 “system extraction failure” in Table 4. In contrast, the lower recall compared to precision for the plan data indicate that cause category 2, which is “incorrect extraction by the system,” was more common. For the objective data, recall showed similar values to precision, which means that false positives and false negatives occurred equally without bias.

### Mismatch Cause Subcategories

On the basis of the features frequently observed in the cause subcategories, this section discusses possible failures when the system is used in practice for analysis of pharmaceutical care records. The discussion here focuses on cause category 1, which was the most common cause of mismatches for subjective data. Table 5 shows typical examples of cause category 1, which was further divided into 17 subcategories, including “verbs, adjectives, and adverbs”; “expressions that are difficult to grasp as diseases or symptoms”; “lists of dosages (medication to be taken as needed)”; “linguistic representation of laboratory values”; and “item names.”

In cause category 1 “system extraction failure,” many extracted terms are categorized as “verbs, adjectives, adverbs” or “expressions that are difficult to grasp as diseases or symptoms.” In “verbs, adjectives, adverbs,” the system was not supposed to extract general terms, such as *sore*, used by patients. The pharmacist receives the patients’ complaints and clinical information and then describes the patient’s condition and other information in objective and assessment columns, replacing them with technical terminology. However, the system’s inability to extract “verbs, adjectives, and adverbs” might cause the pharmacists to overlook symptoms that they did not consider important. Examples of mismatches for extracted terms in the subcategory “expressions that are difficult to grasp as diseases or symptoms” are terms that are related to the disease state but do not directly indicate the disease state, including *normal appetite*, *sleep*, *bowel* *movements*, *renal function*, *hepatic function*, and *blood electrolyte levels* (Table 5). Such normal findings might be missed due to the system’s inability to extract them. One limitation of investigations involving medical records is the inability to determine the actual occurrence of symptoms that are not explicitly documented in the medical records. The extraction of normal findings is also important because the information that “status of symptoms was documented but they did not occur” is expected to increase the reliability of the results of medical record investigation.

### Future Tasks

Not only for cause category 1 but for the other cause categories as well, the cause of the mismatches between the system and the researchers can be explained by one of the following 2 factors: the training data for the system did not contain similar expressions, or there was a difference between the criteria that the system had learned and the criteria that the researchers used in this study. In particular, expressions that are difficult to grasp as diseases or symptoms were the terms that the researchers decided to collect additionally since they were considered important from the pharmacists’ perspective. Therefore, the systems are often constructed without considering them as extraction targets in studies that aim to collect diseases and symptoms in a simple manner. Using the analysis targets in training data should improve the performance of the system. From a medical safety standpoint, overlooking patients’ information is highly detrimental. Therefore, a high recall is preferable, even if precision decreases somewhat. However, recall was significantly lower than precision for the subjective data (precision=0.82; recall=0.32). Therefore, it is critical to improve recall for the subjective data going forward.

Although the SOAP format used in pharmaceutical care records has been the focus of this study, records are sometimes written in SOAP format by other medical staff, including physicians. In this study, we referred to the subjective data in pharmaceutical care records because of the differences in the kind of attention paid to patients’ changes in clinical state depending on the profession. For example, physicians follow up with patients extensively from disease diagnosis to treatment. Nurses provide not only treatment but also daily care for patients during their hospitalization. In contrast, pharmacists conduct follow-up with patients from a pharmacological perspective, which inevitably includes asking about the beneficial and adverse effects of medications. Therefore, it can be inferred that the descriptions contained in the subjective data of pharmaceutical care records differ from those contained in the subjective data of records by other medical staff, despite the fact they are both subjective data. Consequently, to implement a system that can also analyze pharmaceutical care records, it is imperative to study the subjective data of pharmaceutical care records rather than those of other medical staff.

### Limitations

A limitation of this study is the small sample size, consisting only of patients who received CEZ injection at a single institution. When the system is applied to data from different facilities or data of patients who used different drugs, different results might be obtained due to the differences in recording formats, adverse drug effect profiles, characterizations of the patients’ chief complaints, and the perspectives of the health care providers. Furthermore, the number of records that met the eligibility criteria was smaller than the number of records of patients who received CEZ administration, and the records related to CEZ were possibly the subject of analysis in which pharmacists were less able to show their professional competence.

### Future Use

The possibilities for the use of NER in health care are broad and varied, as shown by the various efforts undertaken in previous studies [4-10]. Because pharmaceutical care records contain a large amount of information on adverse drug effects, it should be possible to alert health care professionals when symptoms of possible adverse drug reactions are extracted with reference to the attached document information. Although medical safety must always be ensured in clinical practice, there is a limit to what can be undertaken due to limited human resources and heavy workloads. However, MedNER-J is expected to help medical staff avoid overlooking patients’ symptoms and thereby improve medical safety. MedNER-J showed relatively high performance on assessment data. Therefore, we can easily follow the pharmacist’s assessment of a patient’s clinical status over time by analyzing all the records of the patient. This follow-up allows the pharmacist to collect patient information without overlooking past medical history. Pharmacists can use this information to check whether a patient has a clinical condition that requires discontinuation or dosage adjustment of the prescribed medication. Through these steps, the pharmacist can provide well-prepared patient guidance. Another possibility is to use the results obtained from analyzing large records to investigate the frequency of adverse drug effects or to discover unknown adverse drug effects based on real-world data. When the system is used to identify adverse drug effects, a larger data set than that of this study is required. For example, the frequency of skin symptoms, a common adverse effect with cefazolin administration, is reported to be approximately 0.5%. The sample size required to identify adverse drug effects with a frequency of 0.5% is 4778 patients, assuming a 95% CI with an error of 0.2%. Several innovations are required to achieve this sample size, such as extending the study period and adding participating facilities. More accurate research can be conducted by assuming the incidence of target adverse effects and trying to ensure the sample size in this manner.

Two research tools “FDA Adverse Event Reporting System” and “Japanese Adverse Drug Event Report database” are currently being used for analyzing adverse drug events. Although these databases have several advantages such as large data size and ease of processing due to structured data, they also have the disadvantage of bias in the reporting process. By contrast, pharmaceutical care records have some difficulties in handling due to unstructured data; however, they have no reporting bias and enable epidemiological studies that directly project the clinical practice. Another major advantage of using pharmaceutical care records is potential for real-time monitoring. New discoveries might be obtained from analyzing large amounts of data that were previously unavailable.

### Conclusions

MedNER-J, a system designed to extract information from physicians’ records, was applied to extract data from pharmaceutical care records. The system showed high performance for assessment data and was less reliable for other types of SOAP data. Our results suggest that to apply the system more effectively to pharmaceutical care records, the amount of training data needs to be increased to focus mainly on subjective data, which include patients’ complaints. This study provides the baseline of Japanese pharmaceutical care records analysis.

## Abbreviations

BERT: bidirectional encoder representations from transformers
CEZ: cefazolin sodium
CRF: conditional random fields
*ICD-10*: *International Classification of Diseases, Tenth Revision*
MedDRA: medical dictionary for regulatory activities
MedNER-J: Medical Named Entity Recognition-Japanese
NER: named entity recognition
NLP: natural language processing
P-N: positive-negative
SOAP: subjective, objective, assessment, and plan
